# Evaluating cognitive disturbances as treatment target and predictor of antidepressant action in major depressive disorder: A NeuroPharm study

**DOI:** 10.1038/s41398-022-02240-1

**Published:** 2022-11-08

**Authors:** Vibeke Høyrup Dam, Dea Siggaard Stenbæk, Kristin Köhler-Forsberg, Cheng Ip, Brice Ozenne, Barbara Jacquelyn Sahakian, Gitte Moos Knudsen, Martin Balslev Jørgensen, Vibe Gedsoe Frokjaer

**Affiliations:** 1 Neurobiology Research Unit, Copenhagen University Hospital Rigshospitalet, Copenhagen, Denmark; 2 Faculty of Health and Medical Sciences, University of Copenhagen, Copenhagen, Denmark; 3 Department of Psychology, University of Copenhagen, Copenhagen, Denmark; 4 Department of Clinical Pharmacology, Lundbeck Pharma A/S, Valby, Denmark; 5 Department of Public Health, Section of Biostatistics, University of Copenhagen, Copenhagen, Denmark; 6 Department of Psychiatry, University of Cambridge, Cambridge, UK; 7 Behavioral and Clinical Neuroscience Institute, University of Cambridge, Cambridge, UK; 8 Psychiatric Center Copenhagen, Copenhagen, Denmark

**Keywords:** Depression, Predictive markers

## Abstract

Cognitive disturbances in major depressive disorder (MDD) constitute a critical treatment target and hold promise as an early predictor of antidepressant treatment response; yet their clinical relevance is not fully established. Therefore, we here investigate if (1) cognitive performance improves over the course of antidepressant treatment and (2) cognitive performance at baseline is predictive of antidepressant treatment response. In the NeuroPharm study (clinical trial id: NCT02869035), 92 antidepressant-free patients with a moderate to severe depressive episode were assessed with a comprehensive cognitive test battery including both cold (emotion-independent) and hot (emotion-dependent) tasks. Patients were tested before and after 12 weeks of standard antidepressant treatment with escitalopram in flexible doses of 10–20 mg. Performance improved across most cognitive domains over the course of antidepressant treatment. Notably, these improvements were independent of improvement in mood symptoms, emphasizing that cognitive disturbances are a distinct symptom and therefore treatment target in MDD. Results did not suggest that performance on any single cognitive measure at baseline was associated with later clinical response to antidepressant treatment. However, a small cluster of patients (*N* = 28) with globally disturbed cognition at baseline exhibited poorer clinical response after 8 but not 12 weeks of antidepressant treatment, suggesting that severe cognitive disturbances may delay treatment response. Thus, while pretreatment cognitive performance on individual tests may not be useful as clinical markers of treatment response, profiles capturing performance across different cognitive domains may be useful for stratification of patients with MDD and could be helpful in future intervention trials.

## Introduction

Major Depressive Disorder (MDD) is a highly heterogeneous disorder. Despite decades of effort, we still do not fully understand the etiology behind MDD and it has been suggested that the diagnosis describes several brain pathologies [1]. This may explain why 30–50% of patients do not respond adequately to Selective Serotonin-Reuptake Inhibitors (SSRIs), the standard first-line treatment for moderate to severe MDD [2]. Importantly, for every failed treatment attempt, chances of remission decrease [3] and clinicians, therefore, face an urgent need for new strategies to optimize antidepressant choices early in the course of the illness. Recognizing the complexity and heterogeneity of MDD, research endeavors have shifted from a one-size-fit-all approach towards precision medicine. This requires the identification of biomarkers that can help stratify patients into clinically meaningful subgroups [4]. In recent years, cognitive functions have been highlighted as a promising candidate for monitoring and even predicting treatment response to antidepressant drugs [5, 6]. Cognitive dysfunction is well-documented in MDD and has been shown for both so-called ‘hot’ and ‘cold’ cognitive domains. Cold cognition describes mental processes that occur independently of emotional states and centers around logic and reasoning. Meanwhile hot cognition describes the processing of affective and social stimuli and may include emotional and motivational mental states. Patients with MDD have consistently been shown to exhibit small to moderate deficits in cold cognitive functions such as processing speed, attention, memory, and executive functions [7]. Disturbances in hot cognitive functions including negative affective biases in emotion recognition and disrupted mentalization are also closely associated with depressive psychopathology [8] and have been posited to play a key role in antidepressant drug actions [9]. Impairments in executive functions, and to a lesser extent slowed psychomotor speed, appear to be associated with poor antidepressant treatment outcome [6]. Additionally, results from the large iSPOT-D trial showed that cognitive performance in a small cluster of patients with broad cognitive deficits (i.e., test scores more than 0.5 SD below healthy controls on 11 out of 13 predominantly cold cognitive domains) was predictive of treatment poor response at the study endpoint after eight weeks of treatment with the SSRI escitalopram [10]. Meanwhile, early changes in emotion processing after administration of antidepressant drugs have shown promise as a tool for guiding clinical decision-making in MDD treatment [11]. Apart from their potential as biomarkers, cognitive disturbances also constitute a critical treatment target in MDD [12] as impaired cognition negatively impacts patients’ everyday functioning and contributes to work presenteeism and absenteeism [13, 14]. Antidepressants appear to have a modest positive effect on cold cognition in patients with MDD [15] and hot cognitive processes in both patients and healthy individuals [16]. For example, antidepressants have been shown to normalize brain networks associated with hot cognitive negative affective bias formation in MDD including prefrontal and limbic circuitries [9]. However, cognitive disturbances do not always fully resolve with the remission of core clinical (i.e., mood, energy, hedonia, and somatic) symptoms [17]. For example, a recent meta-analysis reported small to medium impairments in processing speed, learning and memory, attention, and executive functions in remitted patients compared to healthy individuals [18]. Likewise, sustained disturbances in hot cognitive processes have been reported in otherwise remitted patients [8]. Together, these findings indicate that treatment with antidepressants may alleviate some but not all cognitive symptoms in MDD and further point to a dissociation of core depressive symptoms and cognitive symptoms. It is currently unclear to what extent cognitive biomarkers can be used to guide clinical decision making in MDD and to what extent cognitive dysfunction can be rescued by conventional antidepressant treatment. Furthermore, studies investigating both hot and cold cognitive disturbances in MDD are scarce, making it difficult to map and contrast the effect of antidepressant treatment on different types of cognition.

### Aims of the study

We here investigate hot and cold cognitive functioning in a cohort of non-psychotic and antidepressant-free patients before and after 12 weeks of standard serotonergic drug treatment to assess (1) changes in cognitive performance over the course of antidepressant treatment and (2) if cognitive baseline performance can be used to predict treatment response. In addition, we explore the clinical relevance of three distinct clusters of cognitive profiles identified earlier in the present cohort [19].

## Methods

We here report findings from the cognitive part of the NeuroPharm study; a longitudinal, open-label clinical trial investigating potential biomarkers in antidepressant treatment of MDD [20]. The authors assert that all procedures contributing to this work comply with the ethical standards of the relevant national and institutional committees on human experimentation and with the Helsinki Declaration of 1975, as revised in 2008. All procedures involving human patients were approved by the National Committee on Health Research Ethics (H-15017713), the Danish Data Protection Agency (04711/RH-2016-163), the Danish Medicines Agency (2016-001626-34) and pre-registered at https://clinicaltrial.gov (NCT02869035). A detailed description of the full clinical trial including power calculations and main analyses of the cognitive domain is outlined in the published study protocol [20].

### Participants

One hundred patients with MDD were recruited through a central referral site part of the Mental Health Services in the Capital Region of Denmark or through their general practitioner (see Supplementary Materials for CONSORT flow diagram). MDD was diagnosed by a trained clinician in accordance with ICD-10 criteria and confirmed with a Mini-International Neuropsychiatric Interview. To be eligible for inclusion, patients had to be 18–65 years old and have a 17-item Hamilton Depression Rating (HDRS_17_) score >17, indicating a moderate to severe depressive episode. Exclusion criteria included: use of antidepressants within two months of inclusion; more than one antidepressant attempt during the current episode; current episode lasting longer than 2 years; previous non-response or contraindication to SSRIs; current psychotherapeutic treatment; acute suicidal ideation or psychosis; severe somatic illness; history of other primary Axis I psychiatric disorders; substance or alcohol abuse; brain trauma; pregnancy or breastfeeding; and non-fluency in Danish. Written informed consent was obtained from all patients.

### Study program

Out of the 100 patients who entered the study, cognitive data was collected from 92 patients at baseline (67 females). After completing the investigative baseline program, patients started standard antidepressant treatment with 5 mg of escitalopram before increasing to flexible doses of 10–20 mg/day. Dosages were adjusted by trained physicians at follow-up visits at week 1, 2, 4, 8, and 12. In accordance with standard practice, patients experiencing severe side effects or showing poor response to escitalopram after 4 weeks of treatment were offered to switch to the Serotonin-Norepinephrine Reuptake Inhibitor (SNRI) duloxetine (*N* = 16). Cognitive follow-up data was collected after 12 weeks (*N* = 69) and clinical follow-up data was collected after 8 weeks (*N* = 78) and 12 weeks (*N* = 76). Cognitive testing took place in standardized test rooms by trained neuropsychological testers.

#### Clinical outcomes

Depressive symptom severity was assessed with HDRS_17_ and its subscale of 6 items (HDRS_6_, items: depressed mood; guilt; work and interests; psychomotor retardation; psychic anxiety; and somatic symptoms) at baseline and after 4, 8 and 12 weeks of treatment. Primary clinical outcome was categorical treatment status at week 8 classified as either ‘remitter’ and ‘non-responder’. Remitter status was defined by early response (≥50% reduction in HDRS_6_ at week 4) and a HDRS_6_ score below 5 at week 8. Non-responder status was defined by early non-response (<25% reduction in HDRS_6_ score at week 4) and <50% reduction in HDSR_6_ score at week 8. Patients who did not meet criteria for either remitter or non-responder status were classified as ‘intermediate responders’ and were not included in analyses using categorical treatment status as outcome. Secondary clinical outcomes were relative change in HDRS_6_ score (ΔHDRS_6_) calculated as percentage change in HDRS_6_ from baseline to follow-up at week 8 or week 12 and included all patients. Although HDRS_17_ scores are more widely reported in the literature, we choose the HDRS_6_ as it specifically captures core depressive symptoms and has been shown to be more sensitive to antidepressant treatment response [21, 22]. To allow comparisons with other studies, HDRS_17_ results are presented in Supplementary Materials.

#### Cognitive measures

Eleven primary cognitive outcomes were derived from seven neuropsychological tasks capturing both hot and cold cognitive functions with tasks from the hot cognitive domain being further subdivided into an emotion processing bias domain and a social cognitive domain. Emotion processing outcomes included affective bias in emotion recognition (hit rate) and misattribution (false alarm rate) from the *Emotional Recognition Task* (eyes version) [23]; affective bias in emotion detection threshold from the *Emotional Intensity Morphing* task [23]; and affective memory bias from the *Affective Verbal Memory Task 26* (VAMT-26) [24]. Affective bias outcomes were constructed by subtracting negatively valenced scores (e.g., hit rate for sad faces) from positively valenced scores (e.g., hit rate for happy faces) with the exception of the *Intensity Morphing Task* where low scores reflect a better performance and here the affective bias was calculated by subtracting the happy condition from the sad condition: this was done to ensure that the interpretation of the bias scores were consistent across tasks such that a positive bias score reflects preferential processing of positive emotions over negative emotions. The social cognition domain included ratings of guilt and shame from the *Moral Emotions* task and social information preference and social interpretation bias from the *Social Information Preference* task [23]. Lastly, the cold cognitive domain included verbal memory assessed with the VAMT-26; working memory assessed with the *Letter Number Sequence* task; and reaction time assessed with a *Simple Reaction Time* task. A detailed overview of the cognitive tasks and outcomes has been published elsewhere [19].

#### Cognitive profiles clusters

We previously identified three distinct cognitive profiles in the present MDD cohort based on performance across cognitive domains at baseline. The clusters were determined using a data-driven approach where patients’ cognitive baseline scores were z-transformed and a hierarchical cluster algorithm was used to determine the optimal number of clusters and centroids. This information was then used to initializing a K-means cluster analysis that produced the final cognitive profile clusters (see Dam, Stenbæk [19] for a more detailed description). Briefly, Cluster A patients (*n* = 38) were primarily characterized by strong negative affective biases (i.e., tendency to be faster and more accurate at recognizing sad emotions compared to happy emotions) in emotion processing tasks and minimal deficits in cold cognitive domains; Cluster B patients (*n* = 28) were primarily characterized by positive affective biases in emotion processing and moderate deficits in cold cognitive domains; and lastly Cluster C patients (*n* = 26) were characterized by severe global disturbances across all cognitive domains (see Table S1 for a detailed overview).

### Statistical analyses

#### Cognitive performance at baseline and antidepressant treatment response

In a planned analysis, we used logistic regression models to test the association between cognitive performance at baseline and the primary clinical outcome of treatment status (remitter vs non-responder) at week 8. In addition, planned linear regression models were used to test the association between cognitive performance at baseline and ΔHDRS_6_ at week 8 and week 12. Lastly exploratory one-way ANCOVAs were used to assess group difference between the cognitive profile clusters on ΔHDRS_6_ at week 8 and week 12. Age and sex were included in all models.

#### Cognitive change over course of antidepressant treatment

In a planned analysis, we used linear mixed-effect models to assess changes in cognitive performance between baseline and week 12. Age and sex were included in all models.

#### Correlation between change in cognition and change in HDRS_6_ core depressive symptom severity

In an exploratory analysis, we used Spearman’s rank correlation coefficients to assess whether change in cognitive performance was correlated with ΔHDRS_6_ at week 12.

#### Missing data, outliers, and correction for multiple comparisons

Complete case analysis was used to handle missing values in linear, correlation and ANCOVA models while full information maximum likelihood was used in linear mixed effect models (see Fig. S1 for a detailed overview of dropouts). Patients for whom medication compliance could not be confirmed from blood serum levels measured at week 8 were treated as dropouts (*n* = 3). Two outliers were detected for the *Simple Reaction Time* task (7.2 SD and 3.2 SD above the mean): these two datapoints were capped to 2 and 1 millisecond above the third highest score respectively. To control the family-wise error rate, significance thresholds in analyses that included all 11 task outcomes were adjusted for 11 tests using the Bonferroni–Holm method [25].

## Results

Patients were between 18 and 57 years old (mean = 27.3; SD = 8.1). At week 8, 20 patients (25.6%) fulfilled the criteria for remitter status; 44 patients (56.4%) fulfilled the criteria for intermediate responder status, and 14 patients (17.9%) fulfilled the criteria for non-responder status (see Table S1 for additional descriptive details).

### Cognitive performance at baseline and antidepressant treatment response

**Fig. 1:**
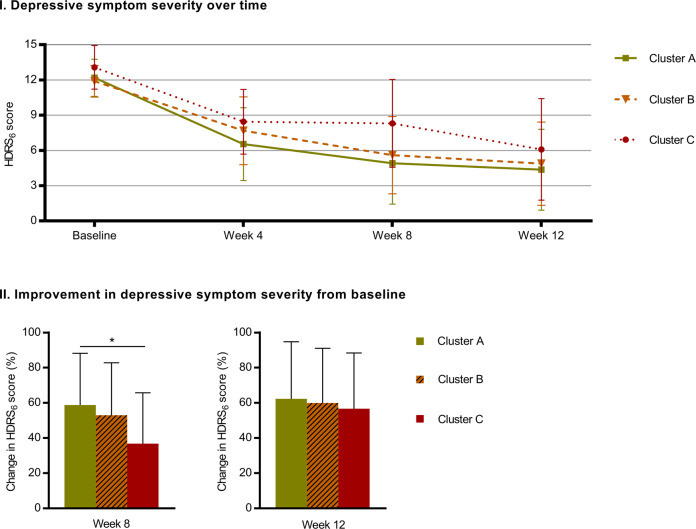
Change in depressive symptom severity over time for cognitive profile clusters. Panel I shows Hamilton Depressive Rating Scale 6 (HDRS_6_) scores for each of the three cognitive profile clusters at baseline, week 4, week 8, and week 12. Panel II shows improvement (i.e., reduction) in HDRS_6_ scores in percentage for the three cognitive profile clusters at week 8 and week 12 relative to baseline. The graphs show observed group averages and the error bars denote standard deviations while the significance notation in panel II represents model estimates corrected for age and sex. * *p* < 0.05.

Table 1 shows associations between cognitive performance at baseline and treatment status (remitter vs non-responder) at week 8 as well as ΔHDRS_6_ for week 8 and week 12. Figure 1 shows absolute and percentage change in HDRS_6_ scores over the course of antidepressant treatment for the three clusters.

**Table 1:** Association between baseline cognition and treatment response.

	Status: remitters vs non-responders				Week 8 ΔHDRS_6_ score			Week 12 ΔHDRS_6_ score		
	OR	OR 95% CI	*p*	*p*_cor_	*β*	*p*	*p*_cor_	*β*	*p*	*p*_cor_
Emotion processing bias										
Recognition bias	1.01	[0.97–1.05]	0.70	1.00	0.02	0.90	1.00	0.02	0.91	1.00
Misattribution bias	0.99	[0.94–1.05]	0.77	1.00	0.15	0.52	1.00	0.22	0.35	1.00
Detection bias	1.00	[0.92–1.08]	0.96	1.00	−0.09	0.71	1.00	0.21	0.42	1.00
Affective memory	1.00	[0.94–1.07]	0.92	1.00	0.20	0.47	1.00	0.19	0.52	1.00
Social cognition										
Guilt ratings	0.50	[0.13–2.04]	0.34	1.00	7.18	0.22	1.00	−3.25	0.59	1.00
Shame ratings	0.93	[0.28–3.12]	0.91	1.00	4.55	0.33	1.00	0.66	0.89	1.00
Information sampling	1.00	[0.96–1.05]	0.84	1.00	0.11	0.60	1.00	0.02	0.91	1.00
Social interpretation bias	1.02	[0.97–1.07]	0.42	1.00	−0.17	0.36	1.00	0.00	1.00	1.00
Cold cognition										
Verbal memory	1.09	[0.87–1.38]	0.46	1.00	−0.70	0.44	1.00	−0.39	0.68	1.00
Working memory	1.02	[0.75–1.37]	0.91	1.00	0.50	0.71	1.00	1.00	0.49	1.00
Reaction time	1.00	[0.98–1.02]	0.99	1.00	0.08	0.20	1.00	0.05	0.46	1.00

Odds ratio (OR) for the treatment status (remitter vs non-responder) at week 8 with respect to cognitive scores measured at baseline (column 2) were estimated using a logistic regression. Also reported are regression coefficients (*β)* for the relative change in HDRS_6_ scores from baseline to week 8 and week 12 by unit of cognitive score at baseline, estimated using a linear regression. Age and sex are included in all models; *p*_cor_ denotes the *p*-values after correction for 11 tests using the Bonferroni–Holm method.

We found no evidence that any individual cognitive task score at baseline was associated with clinical outcome in terms of HDRS_6_ scores after antidepressant treatment. Odds ratios (OR) estimating associations between baseline cognitive performance on individual tasks and treatment status at week 8 were close to 1 (ranging from 0.93 to 1.09; except ratings for guilt, OR = 0.50) and statistically non-significant (all *p*_corrected_ = 1.0). Similarly, we observed no significant association between baseline cognitive performance on individual tasks and ΔHDRS_6_ at week 8 (all *p*_corrected_ = 1.0) or week 12 (all *p*_corrected_ = 1.0). Figure 1 shows absolute and percentage change in HDRS_6_ scores over the course of antidepressant treatment for the three cognitive profile clusters.

At group level, there was a statistically significant difference between the three clusters for ΔHDRS_6_ scores week 8 (*p* = 0.03) but not at week 12 (*p* = 0.8). Follow-up analyses revealed that Cluster C patients had worse response to antidepressant treatment at week 8 than Cluster A patients (*p* = 0.009). Meanwhile, antidepressant dose was similar for three clusters at week 8 (Cluster A = 16.7 mg±3.8; Cluster B = 17.5 ± 3.7; Cluster C = 16.3 ± 4.0).

### Cognitive change over the course of antidepressant treatment

**Fig. 2:**
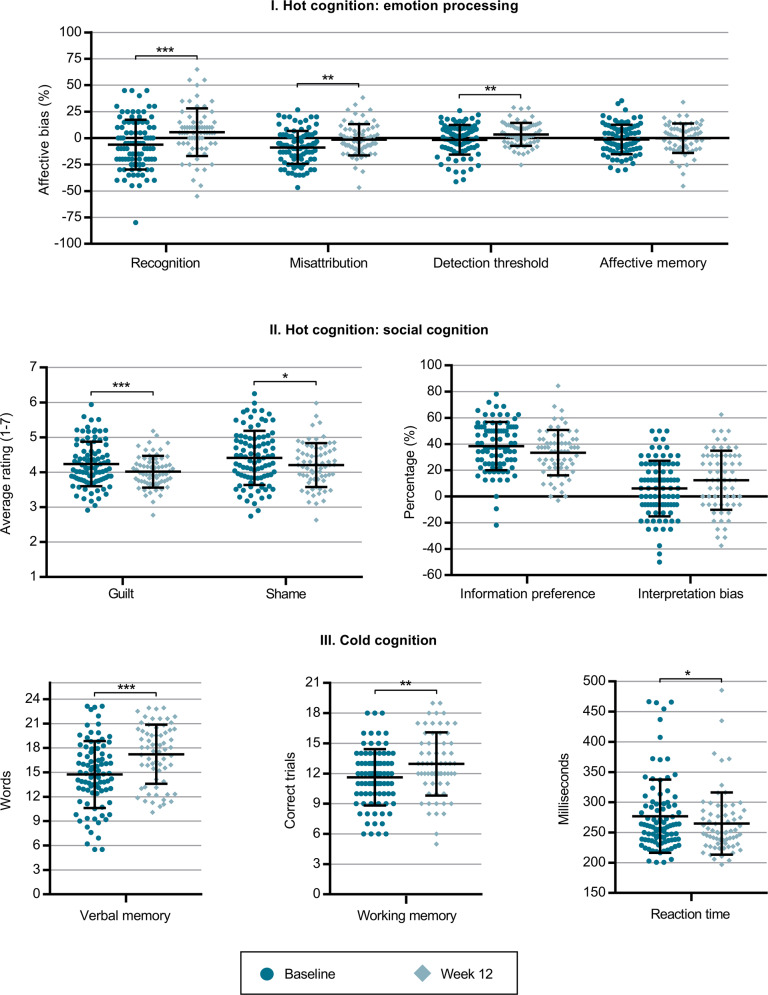
Changes in cognitive performance from baseline to week 12. Changes in affective, social, and cold cognitive outcomes before (*N* = 92) and after 12 weeks (*N* = 69) of antidepressant treatment. Panel I. Affective cognition: *Recognition* = *affective bias for hit rate in the Emotional Recognition Task calculated as hit rate for happy faces minus hit rate for sad faces; Misattribution* = *affective bias for false alarm rate in the Emotional Recognition Task calculated as false alarm rate for happy faces minus false alarm rate for sad faces; Detection threshold* = *affective bias for the Intensity Morphing Task calculated as detection threshold for sad faces minus detection threshold for happy faces; Affective memory* = *affective bias for the Verbal Affective Memory Task 26 calculated as number of remembered positive words minus number of remembered negative words*. Panel II. Social cognition: *Guilt* = *average ratings of guilt in the Moral Emotions task; Shame* = *average ratings of shame in the Moral Emotions task; Information preference* = *choice of theory of mind-related information (thoughts and emotions) relative to facts in the Social Information Preference task; Interpretation bias* = *affective bias in choice of outcome in the Social Information Preference task*. Panel III. Cold cognition: *Verbal memory* = *total recall score for the Verbal Affective Memory Task; Working memory* = *total score in Letter-Number Sequence task; Reaction time* = *Simple Reaction Time*. Note error bars denote standard deviations and individual data points represent observed values while the significance notation represents model estimates corrected for age and sex; *p*-values were corrected for 11 tests using the Bonferroni-Holm method. * *p* < 0.05, ** *p* < 0.01, *** *p* < 0.001.

Figure 2 shows difference in cognitive performance at baseline and after 12 weeks of antidepressant treatment.

### Hot cognition: emotion processing bias

At group level, affective bias for emotion recognition increased (i.e., became more positive) by 11.1 percentage points (95% CI = [6.0;16.3], *p*_corrected_ < 0.001); affective bias for emotion misattribution increased by 7.0 percentage points (95% CI = [3.5;10.6], *p*_corrected_ = 0.002); and affective bias for emotion detection threshold increased by 5.5 percentage points (95% CI = [2.4;8.6], *p*_corrected_ = 0.009). Meanwhile, the increase in affective memory bias was not significant (estimate = 1.7 percentage points, 95% CI = [−2.2;5.7], *p*_corrected_ = 1.0).

### Hot cognition: social cognition

Ratings of negative moral emotions decreased by 0.2 points on a seven-point Likert scale for both guilt (95% CI = [−0.3;−0.1], *p*_corrected_ < 0.001) and shame (95% CI = [−0.4;−0.1], *p*_corrected_ = 0.01). Neither change in preference for social information (estimate = 4.9 percentage points, 95% CI = [−9.6;−0.1], *p*_corrected_ = 0.5) nor change in social interpretation bias (estimate = 5.9 percentage points, 95% CI = [0.5;11.3], *p*_corrected_ = 0.4) were statistically significant.

### Cold cognition

Overall memory capacity increased by 2.6 words (max score 26 words; 95% CI = [2.0;3.2], *p*_corrected_ < 0.001); working memory capacity increased by 1.3 points (max score 21 points; 95% CI = [0.7;1.9], *p*_corrected_ = 0.001); and reaction time improved by 13.7 milliseconds (95% CI = [−22.0;−5.5], *p*_corrected_ = 0.02).

### Correlation between change in cognition and change in HDRS6 core depressive symptom severity

Table 2 shows correlations between changes in cognition between performance from baseline to week 12 and ΔHDRS_6_ at week 12.

**Table 2:** Correlation between change in cognitive score and week 12 ΔHDRS_6_.

	Week 12 ΔHDRS_6_		
	*r*	*p*	*p*_corrected_
Change in emotion processing			
Recognition bias	−0.07	0.57	1.00
Misattribution bias	−0.18	0.14	1.00
Detection bias	−0.04	0.77	1.00
Affective memory bias	−0.11	0.38	1.00
Change in social cognition			
Guilt rating	0.14	0.26	1.00
Shame rating	0.07	0.55	1.00
Information sampling	0.02	0.90	1.00
Social interpretation bias	0.11	0.37	1.00
Change in cold cognition			
Verbal memory	0.08	0.54	1.00
Working memory	−0.04	0.78	1.00
Reaction time	0.04	0.77	1.00

Correlation between changes in cognitive scores from baseline to Week 12 and percentage change in depressive symptom severity from baseline to week 12. Both raw *p*-values and *p*-values corrected for 11 tests using the Bonferroni-Holm method are shown.

Correlations between changes in cognitive performance and week 12 ΔHDRS_6_ were not statistically significantly correlated (all *p*_corrected_ = 1.0) with effect sizes ranging from −0.18 to 0.14.

## Discussion

### Cognitive disturbances as marker of treatment response

We found no evidence that performance on any single cognitive outcome at baseline was associated with remission or non-responder status at week 8. Nor did we observe any significant associations between pre-treatment cognitive performance on individual cognitive tasks and changes in depressive symptoms after 8 or 12 weeks of antidepressant treatment. A recent review found that deficits in cold cognitive domains including executive and psychomotor functions are predictive of antidepressant treatment response in MDD [6]. However, the evidence was only consistent for elderly patients whereas the literature on younger patients was highly conflicted. Together with our negative finding, this suggests that even if a single cognitive function has some predictive value, it is likely too limited to be clinically relevant. Instead, cognitive profiles capturing patterns of performance across several cognitive domains may provide a stronger predictive construct. In the large iSPOT-D trial (baseline, *N* = 1008; completers, *N* = 665), two patient subgroups were identified using cluster analysis: a large group (~75%) with intact cognitive functions and a smaller (~25%) with broadly impaired cognitive functions [10]. Importantly, the study found that the impaired group had poorer clinical response after 8 weeks of antidepressant treatment and that treatment response could be predicted by baseline performance within the impaired group for patients who received treatment with escitalopram [10]. Using a similar data-driven clustering approach, we previously identified three clusters with distinct cognitive profiles in the NeuroPharm cohort [19]. Notably, patients from Cluster C (~28%), who were characterized by severe global cognitive deficits, had poorer clinical treatment response after 8 weeks of serotonergic treatment, mirroring the findings from the iSPOT-D trial. However, this difference in clinical response was not detectable after 12 weeks where Cluster C patients ‘caught up’ to both Cluster A and Cluster B patients. Together, this suggests that while global cognitive impairments may slow or delay treatment response, it does not necessarily affect longer-term treatment outcome. This is important because current guidelines suggest that patients should be switched to a different antidepressant drug if adequate treatment response is not observed within 4–8 weeks [26]. One implication is therefore that clinicians might consider waiting longer to assess the effect of the first-line antidepressant before switching medication for this group of patients or consider starting patients with severe global cognitive deficits on other potentially more potent treatment regiments.

### Changes in cognitive performance over the course of antidepressant treatment

We found significant improvements in both hot and cold cognitive domains after 12 weeks of serotonergic treatment. This included increases in positive bias for emotion recognition, misattribution, and detection; decreased ratings of guilt and shame; improved verbal and working memory; and faster reaction time. Meanwhile, there was no significant change for affective bias in verbal memory; social information preference; or social interpretation bias. This is perhaps not surprising, as we did not observe any disturbances on these task outcomes when we compared the same cohort of MDD patients with healthy controls at baseline [19], suggesting that they may not be sensitive and/or relevant to MDD pathology. Overall, our findings align with previous reports that antidepressant treatment improve cognition across both hot [16] and cold domains [15].

### Dissociation between cognitive disturbances and core depressive symptoms in MDD

We observed no statistically significant correlation between improvements in core depressive symptoms and changes in cognitive performance over the course of antidepressant treatment. As we also did not observe an association between cognitive performance and depression severity at baseline [19], we argue that disturbed cognition should be seen as a distinct symptom in depression and not merely as an epiphenomenon (i.e., extension) of mood and somatic symptoms. This is supported by other large clinical studies which also found no or only partial overlap between treatment effects on cognition and core depressive symptoms [27, 28]. Consequently, this interpretation raises the intriguing possibility of parallel mechanisms of drug action for cognitive and mood symptom modalities in MDD, which would need to be verified in future studies. Meanwhile, a contrasting view of antidepressant drug action in MDD is offered by the cognitive neuropsychological model of depression [9]. The model posits that antidepressant drugs act by acutely remediating negative affective biases. Over time, the changes in affective biases enable positive restructuring of dysfunctional cognitive processes, which ultimately leads to alleviation of mood symptoms [29]. While not in direct conflict with the prediction that early improvements in affective biases predict later treatment response, our observation that longer-term improvements in affective biases are *not* related to clinical improvement does not lend support to the cognitive neuropsychological model of depression. Rather, our findings suggest that the interaction between cognition and core depressive symptoms over the course of antidepressant treatment may be more complex than previously thought.

### Methodological limitations

The present findings should be interpreted in the light of several limitations. First, the present study did not include a healthy control group and/or placebo group. This means that we cannot account for any potential learning effect in cognitive performance and may consequently be overestimating antidepressant effects on cognition. While this is likely not an issue for the hot cognitive domains, as none of the chosen task outcomes included learning aspects [23], the cold cognitive domains, and in particular the verbal memory task, are more vulnerable to effects of repeated testing. In addition, the study design did not allow us to investigate early changes (1–2 weeks) in cognitive performance as a predictor of long-term clinical outcomes despite both theoretical [9] and recent empirical [30, 31] support for such an association. Potential limitations of the included tasks should also be considered as it cannot be ruled out that different or more difficult tasks, particularly within the cold cognitive domain, may be more sensitive markers of antidepressant treatment response. Importantly, as we investigated the effects of escitalopram (and duloxetine as a second line treatment), our findings may not be generalizable to treatments with other antidepressants drugs. Lastly, the included patients we predominantly young and female and we did not account for potential effects of education level which may reduce the generalizability of our findings for patient groups with different demographic profiles, e.g., older populations.

### Implications and future perspectives

Our findings emphasize not only the complexity of cognitive disturbances in depression but also their importance as a distinct symptom and therefore treatment target in MDD. Our findings further show that while pretreatment cognitive performance on individual tests may not be clinically useful as markers of treatment response, cognitive profiles that map performance across a range of cognitive domains may be useful stratification tools in MDD. For example, our findings suggest that conventional antidepressant treatment response is delayed in patients with global cognitive disturbances which could impact clinical treatment choices. Importantly, this should also inform future studies to investigate possible disease mechanisms including whether such cognitive profiles relate to biological phenotypes (e.g., neurophysiological or neurochemical characteristics) and whether patients with a certain profile may respond better to specific antidepressant drugs or non-pharmacological treatments. It would also be relevant to investigate whether early treatment response may be improved in patients with global dysfunction through antidepressant treatment augmented with cognitive remediation training or cognition-enhancing drugs [32].

## Supplementary information

Supplementary Materials
