# Supplementary material for: Evaluating cognitive disturbances as treatment target and predictor of antidepressant action in major depressive disorder: A NeuroPharm study
Source: Transl Psychiatry. 2022 Nov 8;12:468. doi: 10.1038/s41398-022-02240-1 (PMC9643376; doi:10.1038/s41398-022-02240-1)
Supplement: Supplementary file 1 — Supplementary Materials [file 41398_2022_2240_MOESM1_ESM.docx]

**Supplementary Materials**

1. Consort Flow Diagram for NeuroPharm clinical trial…...…………………………………… p. 2
2. Overview of cognitive profile clusters……………………………………………………….. p. 3
3. Descriptive information for patient cohort over trial period…………………………………. p. 4
4. Depressive symptom severity across clusters………………………………………………… p. 5
5. Correlation between changes in cognition and changes in clinical symptoms………………. p. 6
6. Changes in cognitive performance from baseline to week 12 across clusters……………….. p. 7

**1. Consort Flow Diagram for NeuroPharm clinical trial**

Figure S1. Consort flow diagram for NeuroPharm clinical trial


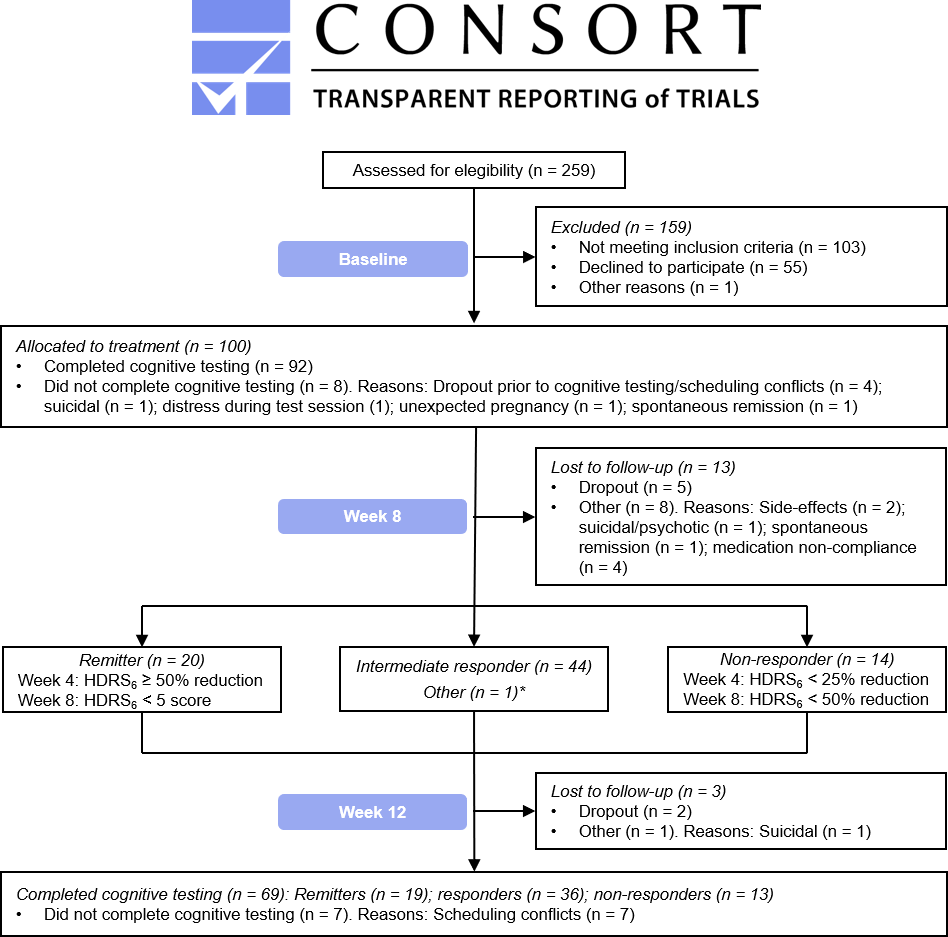


**Figure S1.** Consort flow showing enrollment of patients, treatment status and drop-outs throughout the NeuroPharm clinical trial. *One patient missed their clinical assessment at week 8 and could therefore not be classified into any of the three treatment status categories.

**2. Overview of cognitive profile clusters**

The three cognitive profile clusters were previously identified using a combination of Hierarchical Cluster Analysis and K-means cluster analysis ([1](#_ENREF_1)). They were derived from patient baseline scores on emotion recognition and misattribution from the *Emotional Recognition Task*-eyes version, detection threshold from the *Intensity Morphing* task, guilt and shame ratings from the *Moral Emotions* task, verbal memory from the *Verbal Affective Memory Task-26*, working memory from the *Letter Number Sequence* task, and reaction time from the *Simple Reaction Time* task. Table S1 show average scores on all cognitive outcomes at baseline for each of the three clusters.

| Table S1. Cognitive cluster scores | | | |  |  |  |  |  |  |
| --- | --- | --- | --- | --- | --- | --- | --- | --- | --- |
|  |  | Cluster A (N = 38) | |  | Cluster B (N = 28) | |  | Cluster C (N = 26) | |
|  |  | Mean ± SD | Range |  | Mean ± SD | Range |  | Mean ± SD | Range |
| *Hot cognition: affective biases* | | | |  |  |  |  |  |  |
| Emotion recognition |  | -20.7 ± 16.9 | -80.0–15.0 |  | 18.8 ± 14.9 | -10.0–45.0 |  | -11.7 ± 17.4 | -40.0–20.0 |
| Emotion misattribution |  | -17.6 ± 11.6 | -46.7–3.3 |  | 7.9 ± 10.2 | -13.3–26.7 |  | -14.0 ± 10.7 | -35.0–6.7 |
| Emotion detection |  | 0.5 ± 12.6 | -32.1–19.6 |  | 1.5 ± 10.4 | -17.9–17.9 |  | -8.2 ± 17.3 | -41.4–25.9 |
| Affective memory |  | 1.6 ± 14.8 | -24.0–32.7 |  | -4.9 ± 14.9 | -30.7–35.3 |  | -1.3 ± 10.9 | -18–19.3 |
| *Hot cognition: social cognition* | | | |  |  |  |  |  |  |
| Guilt ratings |  | 3.9 ± 0.4 | 3.1–4.9 |  | 4.0 ± 0.5 | 2.9–5.0 |  | 5.0 ± 0.4 | 4.0–5.9 |
| Shame ratings |  | 4.1 ± 0.6 | 2.7–5.0 |  | 4.0 ± 0.5 | 2.9–5.1 |  | 5.3 ± 0.5 | 4.1–6.3 |
| Information sampling |  | 38.5 ± 18.1 | -21.9–78.1 |  | 36.7 ± 18.3 | 0.0–68.8 |  | 40.3 ± 19.6 | -9.4–71.9 |
| Social interpretation bias |  | 4.0 ± 22.2 | -50.0–37.5 |  | 10.3 ± 16.9 | -18.8–50 |  | 4.5 ± 24.0 | -37.5–50.0 |
| *Cold cognition* |  |  |  |  |  |  |  |  |  |
| Verbal memory |  | 16.8 ± 3.2 | 9.8–23.1 |  | 14.3 ± 3.6 | 6.2–23.0 |  | 12.3 ± 4.4 | 5.5–23.1 |
| Working memory |  | 12.5 ± 2.8 | 8.0–18.0 |  | 11.6 ± 2.5 | 6.0–15.0 |  | 10.5 ± 2.8 | 6.0–16.0 |
| Reaction time |  | 267.4 ± 49.5 | 200.5–454.7 |  | 278.8 ± 57.8 | 219.3–436.9 |  | 288.9 ± 76.9 | 205.4–466.7 |

**Table S1.** Average scores on cognitive outcomes at baseline for the three cognitive profile clusters. Mean, SD and range are shown.

**3. Descriptive information for patient cohort over trial period**

Table S2 shows descriptive and demographic information for the study cohort at different timepoints across the NeuroPharm trial period.

| Table S2. Descriptive factors | |  |  |  |  |  |  |  | |  |  |  |  |
| --- | --- | --- | --- | --- | --- | --- | --- | --- | --- | --- | --- | --- | --- |
|  | Baseline (N = 92) | |  | Week 8 (N = 78) | |  | Week 12 (N = 76)^a^ | | |  | Week 12 (N = 69)^b^ | |  |
|  | N (%) | |  | N (%) | |  | N (%) | | |  | N (%) | |  |
| Sex (female) | 67 (72.8%) | |  | 57 (72.2%) | |  | 54 (71.1%) | | |  | 49 (71.0%) | |  |
| First episode | 41 (44.6%) | |  | 33 (41.8%) | |  | 30 (39.5%) | | |  | 29 (42.0%) | |  |
|  | Mean ± SD | Range |  | Mean ± SD | Range |  | Mean ± SD | | Range |  | Mean ± SD | Range |  |
| Age | 27.3 ± 8.1 | 18–57 |  | 27.5 ± 8.6 | 18-57 |  | 27.4 ± 8.3 | | 18–57 |  | 27.2 ± 8.0 | 18–57 |  |
| HDRS_6_ score | 12.3 ± 1.6 | 7–17 |  | 6.0 ± 3.8 | 0–16 |  | 4.9 ± 3.8 | | 0–14 |  | 4.9 ± 3.7 | 0–14 |  |
| HDRS_17_ score | 22.8 ± 3.4 | 18–31 |  | 11.7 ± 6.6 | 1–31 |  | 9.6 ± 6.1 | | 0–25 |  | 9.4 ± 5.9 | 0–25 |  |
| **Table S2.** Age, sex, patients with first episode depression (vs recurrent), and depressive symptoms severity indexed with Hamilton Depressive Rating Scale 6 (HDRS_6_) and 17 (HDRS_17_). ^a^ All patient with clinical depression score available for week 12. ^b^ Subgroup of patients who completed cognitive testing at week 12. | | | | | | | | | | | | |  |
|  |  |  |  |  |  |  |  |  |  |  |  |  |  |

**4. Depressive symptom severity across clusters**

Table S3 shows clinical depression scores indexed with the Hamilton Depressive Rating Scale 6 (HDRS_6_) and 17 (HDRS_17_) across different timepoints in the NeuroPharm trial.

| Table S3. Cognitive profile clusters and depressive symptoms | | | | | | | | | |  |  |  |  |  |
| --- | --- | --- | --- | --- | --- | --- | --- | --- | --- | --- | --- | --- | --- | --- |
|  | Cluster A | |  | Cluster B | |  | Cluster C | | *p* | A vs B |  | A vs C |  | B vs C |
|  | Mean ± SD | N |  | Mean ± SD | N |  | Mean ± SD | N |  | *p* |  | *p* |  | *p* |
| Baseline HDRS_6_ | 12.2 ± 22.1 | 38 |  | 11.9 ± 1.3 | 28 |  | 13.1 ± 1.9 | 26 | 0.017 | 0.40 |  | 0.037 |  | 0.010 |
| Baseline HDRS_17_ | 22.1 ± 3.2 | 38 |  | 23.0 ± 3.1 | 28 |  | 23.6 ± 3.8 | 26 | 0.19 | 0.29 |  | 0.078 |  | 0.28 |
| Week 8 ΔHDRS_6_ (%) | 58.8 ± 29.4 | 34 |  | 53.0 ± 29.8 | 23 |  | 36.8 ± 29.0 | 22 | 0.029 | 0.56 |  | 0.0087 |  | 0.093 |
| Week 8 ΔHDRS_17_ (%) | 55.1 ± 29.3 | 34 |  | 50.4 ± 24.5 | 23 |  | 37.2 ± 28.8 | 22 | 0.072 | 0.66 |  | 0.032 |  | 0.12 |
| Week 12 ΔHDRS_6_ (%) | 62.3 ± 32.5 | 33 |  | 60.0 ± 31.0 | 22 |  | 56.7 ± 31.6 | 21 | 0.77 | 0.99 |  | 0.49 |  | 0.62 |
| Week 12 ΔHDRS_17_ (%) | 60.6 ± 27.8 | 33 |  | 54.1 ± 28.8 | 22 |  | 54.3 ± 26.2 | 21 | 0.64 | 0.61 |  | 0.37 |  | 0.88 |
| **Table S3.** Hamilton Depressive Rating Scale 6 (HDRS_6_) and 17 (HDRS_17_) scores at baseline and percentage change (i.e. improvement) in HDRS_6/17_ scores after 8 and 12 weeks of antidepressant treatment for the three cognitive profile clusters. ANCOVA models corrected for age and sex were used to determine main effect of groups; also shown are follow-up analyses investigating differences between individual clusters. Note, *p*-values are not corrected for multiple comparisons. | | | | | | | | | | | | | | |

**5. Correlation between changes in cognition and changes in clinical symptoms**

Table S4 shows correlation between absolute change scores on cognitive outcomes and percentage change in HDRS_17_ scores after 12 weeks of antidepressant treatment.

| Table S4. Correlation between change in cognitive score and week 12 ΔHDRS_17_ | | | |
| --- | --- | --- | --- |
|  | Week 12 ΔHDRS_17_ | | |
|  | *rho* | *p* | *p_corrected_* |
| *Hot cognition: affective biases* |  |  |  |
| ΔEmotion recognition | -0.07 | 0.57 | 1.00 |
| ΔEmotion misattribution | -0.18 | 0.14 | 1.00 |
| ΔEmotion detection | 0.03 | 0.81 | 1.00 |
| ΔAffective memory | -0.14 | 0.26 | 1.00 |
| *Hot cognition: social cognition* |  |  |  |
| ΔGuilt rating | 0.06 | 0.63 | 1.00 |
| ΔShame rating | 0.01 | 0.90 | 1.00 |
| ΔInformation sampling | -0.02 | 0.88 | 1.00 |
| ΔSocial interpretation bias | 0.18 | 0.16 | 1.00 |
| *Cold cognition* |  |  |  |
| ΔVerbal memory | 0.07 | 0.60 | 1.00 |
| ΔWorking memory | -0.03 | 0.83 | 1.00 |
| ΔReaction time | 0.06 | 0.60 | 1.00 |
| **Table 2.** Correlation between absolute changes in cognitive scores from baseline to Week 12 and percentage change in Hamilton Depressive Rating Scale 17 (HDRS_17)_ scores from baseline to week 12. Both raw *p*-values and *p*-values corrected for 11 tests using the Bonferroni-Holm method are shown. | | | |

**
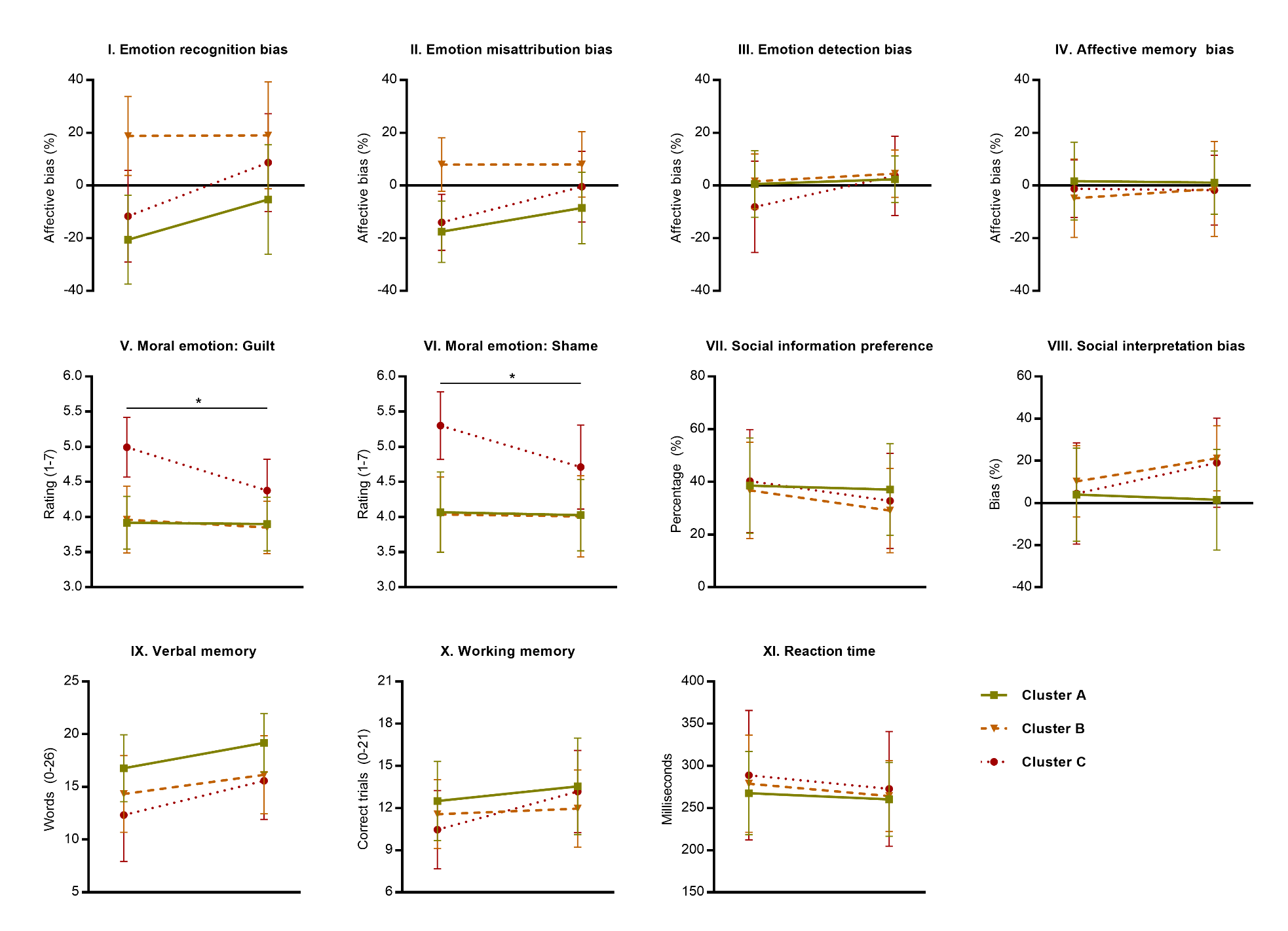
6. Changes in cognitive performance from baseline to week 12 across clusters**

**Fig S2.** Difference in magnitude of changes in cognitive performance from baseline to week 12 for the three cognitive clusters. The graphs show observed group averages at baseline and follow-up at week 12 and the error bars denote standard deviations while the significance notation represents model estimates corrected for age and sex; *p­*-values were corrected for 11 tests using the Bonferroni-Holm method. * *p*<0.05. Significant main effect of changes in cognitive performance was observed for guilt and shame ratings in the *Moral Emotions task* (graph V and VI).

Linear mixed-effect models models showed that at group level, the three cognitive profile clusters differed significantly on changes in ratings of guilt (*p_corrected_*<0.001) and shame (*p_corrected_*=0.008). Follow-up analysis indicated that Cluster C patients experienced a bigger decrease in guilt ratings compared with both Cluster A (*p*<0.001) and Cluster B (*p*=0.002) patients. Similarly, Cluster C patients experienced a bigger decrease in shame ratings than Cluster A (*p*<0.001) and Cluster B (*p*=0.004) patients. Meanwhile, trends for group level differences in cognitive change over time were observed for several other cognitive outcomes including emotion recognition bias (*p*=0.02, *p**_corrected_*=0.2), emotion misattribution bias (*p*=0.02, *p_corrected_*=0.3), emotion detection bias (*p*=0.2, *p _corrected_*=0.3) and working memory (*p*=0.04, *p_corrected_*=0.4).

**References**

1. Dam VH, Stenbæk DS, Köhler-Forsberg K, Ip C, Ozenne B, Sahakian BJ, et al. Hot and cold cognitive disturbances in antidepressant-free patients with major depressive disorder: a NeuroPharm study. Psychological Medicine. 2020:1-10.
